# SG-APSIC1011: Factors associated with improved knowledge of COVID-19 prevention and control following a training of healthcare workers in Vietnam

**DOI:** 10.1017/ash.2023.33

**Published:** 2023-03-16

**Authors:** Hoang Nguyen, Tran Minh Dien, Le Thi Anh Thu, Le Kien Ngai, Pham Thanh Thuy, Do Minh Loan, Ta Anh Tuan, Do Thien Hai, Phan Huu Phuc, Tran Huu Luyen, Huynh Minh Tuan, Le Thi Thanh Thuy, Nguyen Thi Thanh Ha, Bui Nghia Thinh, Do Quoc Huy, Todd M Pollack

**Affiliations:** The Partnership for Health Advancement in Vietnam, Beth Israel Deaconess Medical Center, Hanoi, Vietnam; Vietnam National Children’s Hospital, Hanoi, Vietnam; Ho Chi Minh City Infection Control Society, Ho Chi Minh City, Vietnam; Vietnam National Children’s Hospital, Hanoi, Vietnam; The Partnership for Health Advancement in Vietnam, Beth Israel Deaconess Medical Center, Hanoi, Vietnam; Vietnam National Children’s Hospital, Hanoi, Vietnam; Vietnam National Children’s Hospital, Hanoi, Vietnam; Vietnam National Children’s Hospital, Hanoi, Vietnam; Vietnam National Children’s Hospital, Hanoi, Vietnam; Thua Thien Hue Society of Infection Control, Thua Thien Hue, Vietnam; Ho Chi Minh City Infection Control Society, Ho Chi Minh City, Vietnam; Ho Chi Minh City Infection Control Society, Ho Chi Minh City, Vietnam; Ho Chi Minh City Infection Control Society, Ho Chi Minh City, Vietnam; Thu Duc District Hospital, Ho Chi Minh City, Vietnam; People’s Hospital 115, Ho Chi Minh City, Vietnam; The Partnership for Health Advancement in Vietnam, Beth Israel Deaconess Medical Center, Hanoi, Vietnam

## Abstract

**Objectives:** SARS-CoV-2 is a novel and highly infectious virus. An effective response requires rapid training of healthcare workers (HCWs). We measured the change in knowledge related to COVID-19 and associated factors before and after training of HCWs in Vietnam. **Methods:** A quasi-experimental design was used to evaluate HCW knowledge related to prevention and control of SARS-CoV-2 before and after attending a 2-day training-of-trainers course. Between June and September 2020, 963 HCWs from 194 hospitals in 21 provinces received the training. HCW knowledge was assessed using a 20-item questionnaire consisting of multiple-choice questions at the beginning and closing of the training course. A participant received 1 point for each correct answer. He or she was considered to have improved knowledge the posttest score was higher than the pretest score with a score ≥15 on the posttest. We applied the McNemar test and logistic regression model to test the level of association between demographic factors and change in knowledge of COVID-19. **Results:** Overall, 100% of HCWs completed both the pretest and posttest. At baseline, only 14.7% scored ≥15. Following the training, 78.4% scored ≥15 and 64.3% had improved knowledge according to the predetermined definition. Questions related to the order of PPE donning and doffing and respiratory specimen collection procedures were identified as having the greatest improvement (44.6% and 60.7%, respectively). Being female (OR, 1.5; 95% CI, 1.1–2.0), having a postgraduate degree (OR, 2.5; 95% CI, 1.4–4.4), working in a nonmanager position (OR, 1.5; 95% CI, 1.1–2.1), previous contact with a COVID-19 patient (OR, 1.5; 95% CI, 1.1–2.0), and working in northern Vietnam (OR, 2.0; 95% CI, 1.4–2.6), were associated with greater knowledge improvement. **Conclusions:** Most HCWs demonstrated improved knowledge of COVID-19 prevention and control after attending the training. Particular groups may benefit from additional training: those who are male, leaders and managers, those who hold an undergraduate degree, and those who work in the southern provinces.

